# Cholesterol Reprograms Oxysterol Metabolism via the LOX1/CH25H/CYP7B1 Signaling Axis to Drive Multidrug Resistance in Colorectal Cancer

**DOI:** 10.1155/humu/3675889

**Published:** 2026-04-25

**Authors:** Haixia Cheng, Luyao Huang, Jieshen Huang, Jiasheng Feng, Kehua Huang, Mei Liu, Ji Li

**Affiliations:** ^1^ Laboratory of Cell and Molecular Biology and State Key Laboratory of Molecular Oncology, National Cancer Center/National Clinical Research Center for Cancer/Cancer Hospital, Chinese Academy of Medical Sciences and Peking Union Medical College, Beijing, China, cacms.ac.cn; ^2^ Department of Gastrointestinal Surgery, Shanxi Bethune Hospital, Shanxi Academy of Medical Sciences, Tongji Shanxi Hospital, Third Hospital of Shanxi Medical University, Taiyuan, China; ^3^ School of Medicine, Tongji University, Shanghai, China, tongji.edu.cn; ^4^ Guigang Hospital, Guangxi Medical University, Guangxi, China, gxmu.edu.cn; ^5^ Department of Pediatric Surgery, Shenzhen Nanshan People′s Hospital, Affiliated Nanshan Hospital of Shenzhen University, Shenzhen, China

**Keywords:** CH25H/CYP7B1 axis, chemoresistance, cholesterol, colorectal cancer, metabolic reprogramming

## Abstract

Chemotherapeutic resistance remains a major contributor to tumor recurrence and unfavorable clinical outcomes in colorectal cancer (CRC). Although cholesterol metabolic reprogramming has been implicated in tumorigenesis, metastasis, and drug resistance across multiple malignancies, its specific role in CRC chemoresistance requires systematic investigation. We analyzed RNA‐seq data from GEO dataset GSE196900 to identify differentially expressed genes (|Log2FC| ≥ 1.5, adjusted *p* < 0.05). Functional enrichment analysis (GO/KEGG), protein–protein interaction (PPI) network construction, and gene set enrichment analysis (GSEA) were performed. Experimental validation using 5‐fluorouracil (5‐FU)–resistant CRC cell lines (HCT8/HCT15) included cholesterol/25‐hydroxycholesterol (25‐HC) treatments, assessed through CCK‐8 proliferation assays, wound healing migration tests, quantitative real‐time PCR (qRT‐PCR), Western blotting, and cholesterol metabolite quantification. Integrative bioinformatics and experimental evidence revealed that 5‐FU–resistant CRC cells demonstrate significant upregulation of cholesterol metabolism regulators, including lectin‐type oxidized LDL Receptor 1 (LOX1), cholesterol 25‐hydroxylase (CH25H), and Cytochrome P450 Family 7 Subfamily B Member 1 (CYP7B1). These cells exhibited impaired cholesterol efflux capacity and consequent intracellular cholesterol accumulation. Exogenous supplementation with cholesterol or 25‐HC promoted proliferation, migration, and chemoresistance in both parental and resistant cells. Conversely, CH25H knockdown in resistant cells significantly attenuated malignant phenotypes and restored drug sensitivity. Our findings establish cholesterol metabolic dysregulation as a novel mechanistic contributor to 5‐FU resistance in CRC, mediated through the LOX1‐CH25H‐CYP7B1 regulatory axis. These results propose that therapeutic targeting of cholesterol homeostasis may overcome chemoresistance and improve clinical management of refractory CRC patients.

## 1. Introduction

CRC has emerged as a highly aggressive and life‐threatening malignancy, posing substantial challenges to patient survival and treatment outcomes [1, 2]. Globally, it ranks as the third most commonly diagnosed cancer and the fourth leading cause of cancer‐related deaths, with projections indicating a 60% increase in incidence by 2030 [3]. Current CRC management primarily involves surgical resection combined with chemotherapy, among which 5‐FU serves as a cornerstone agent in most therapeutic protocols [4, 5]. However, the development of chemoresistance—particularly to conventional drugs like 5‐FU and docetaxel—has led to a progressive decline in treatment efficacy [6].

Thus, there is an urgent need to identify novel molecular targets capable of overcoming therapeutic resistance and improving patient survival. Metabolic reprogramming represents a hallmark of cancer, critically influencing tumor progression and treatment response [7]. Neoplastic cells alter their metabolism of glucose, amino acids, and lipids to support rapid proliferation and survival [8, 9]. In CRC, multiple metabolic pathways are dysregulated compared with normal mucosa. For instance, Bohn et al. reported that enhanced glycolysis acidifies the tumor microenvironment (TME), activating G protein–coupled receptor signaling and promoting transcriptional reprogramming that fuels CRC growth [10]. Bertero et al. observed that cancer‐associated fibroblasts (CAFs) migrate toward regions rich in glutamine, enhancing invasiveness and therapy resistance [11]. Moreover, Ringel et al. demonstrated that high‐fat diets exacerbate CRC progression by impairing CD8+ T cell infiltration and antitumor immunity [12]. Lipid metabolic rewiring is increasingly recognized as a pivotal event in cancer, influencing the immune landscape and clinical prognosis of CRC [8, 13]. Targeting key lipid metabolic enzymes and pathways may thus represent a promising strategy for restoring chemosensitivity [14].

As a key lipid component, cholesterol is essential for cellular integrity and systemic homeostasis, participating in immune activation and bile acid synthesis that modulates intestinal immunity [8]. To sustain rapid proliferation, cancer cells upregulate cholesterol acquisition and synthesis, facilitating membrane biogenesis and signaling [15]. Altered cholesterol metabolism has been implicated in the progression of multiple cancers, including those of the liver, prostate, breast, and colorectum [15, 16]. Wang et al. demonstrated enhanced cholesterol biosynthesis in murine intestinal tumor models [17]. Jacobs et al. linked the mevalonate pathway—a key cholesterol synthesis route—to increased CRC risk [18]. Collectively, these studies indicate that cholesterol synthesis and uptake contribute to CRC proliferation, migration, and metastasis. Nonetheless, the potential role of cholesterol in mediating 5‐FU resistance in CRC remains inadequately explored.

In this study, we integrated bioinformatic analyses with experimental validation to investigate the involvement of cholesterol metabolism in 5‐FU–resistant CRC. Our results indicate that cholesterol levels are elevated in chemoresistant CRC cells and contribute to their resistant phenotype. These findings highlight the therapeutic potential of modulating cholesterol metabolism to counteract chemoresistance in CRC.

## 2. Materials and Methods

### 2.1. Data Preparation and Identification of Differentially Expressed Genes (DEGs)

Gene expression datasets related to drug‐resistant CRC were retrieved from the Gene Expression Omnibus (GEO) database (https://wwwncbi.nlm.nih.gov/geo) using the search terms “colorectal cancer” and “drug resistant.” The dataset GSE196900, which profiles drug‐resistant CRC cell lines, was selected for analysis. DEGs were identified based on an adjusted *p* value < 0.05 and an absolute log2 fold change (|Log2FC|) ≥ 1.5. Volcano plots and heatmaps were generated using R software [19].

### 2.2. Enrichment Analysis

Functional enrichment analyses were conducted using the DAVID bioinformatics platform (https://davidbioinformatics.nih.gov). Gene Ontology (GO) terms—including biological process (BP), molecular function (MF), and cellular component (CC)—and Kyoto Encyclopedia of Genes and Genomes (KEGG) pathways were analyzed. Terms with a *p* value < 0.05 were considered statistically significant. Gene set enrichment analysis (GSEA) was additionally performed (https://www.gsea-msigdb.org/gsea) to identify potential signaling pathways and biological processes.

### 2.3. Construction of the PPI Network

A PPI network was constructed using the STRING database (https://string-db.org), with *Homo sapiens* set as the reference organism. Interactions between disease‐related and drug‐target proteins were mapped, and the resulting network was visualized in STRING. Node size and color were used to represent the strength of protein interactions.

### 2.4. Cell Culture

Human CRC cell lines HCT15 (RRID: CVCL_0292) and HCT8 (RRID: CVCL_2478) were obtained from the Shanghai Institute of Biochemical Cell Science, Chinese Academy of Sciences. All cells tested negative for mycoplasma contamination throughout the study. Cells were maintained in high‐glucose Dulbecco′s Modified Eagle Medium (DMEM) (Gibco, 11995065) supplemented with 10% fetal bovine serum (FBS) (Sciencell, 0500) and 1% penicillin–streptomycin (HyClone, SV30010). The medium was refreshed every 2 days, and cells were passaged at 80%–90% confluence. For cholesterol supplementation experiments, various concentrations of cholesterol were added to the DMEM. 5‐FU was also introduced to assess drug resistance.

### 2.5. siRNA‐Mediated Knockdown

Cells were seeded into 10‐cm dishes with 15 mL of antibiotic‐free medium. After 24 h, 180 pmol of siRNA (Thermo Fisher Scientific) was mixed with 1.5‐mL Opti‐MEM I Medium (Thermo Fisher Scientific) and incubated at room temperature for 20 min before being added to the cells. The following day, cells were harvested and plated into six‐well plates for subsequent experiments. Knockdown efficiency was evaluated by measuring target gene expression.

### 2.6. Cell Proliferation and Migration

Cells were seeded into 96‐well plates and treated with varying concentrations of cholesterol for 24 and 48 h. Cell viability was assessed using a Cell Counting Kit‐8 (CCK8) (Biosharp, BS350A), and absorbance was measured at 450 nm using a microplate reader. For migration analysis, wound healing assays were performed. HCT15 and HCT8 cells were grown in 6‐well plates and treated with 100‐*μ*M butyrate or vehicle control for 24 h. Wound closure was quantified using ImageJ software. All experiments were performed in triplicate.

### 2.7. Measurement of Cholesterol, 25‐Hydroxycholesterol (25‐HC), and 7*α*,25‐Dihydroxycholesterol (7*α*,25‐HC) Concentration

Intracellular levels of cholesterol, 25‐HC, and 7*α*,25‐HC were measured. Briefly, cells were washed with cold PBS and lysed in RIPA buffer containing protease inhibitors. Lipids were extracted using a chloroform: methanol (2:1, *v*/*v*) mixture. After centrifugation at 3000 rpm for 10 min at 4°C (DT5‐4B centrifuge, Beili Co., Beijing), the organic phase was collected and dried under nitrogen. The residue was reconstituted in assay buffer, and cholesterol, 25‐HC, and 7*α*,25‐HC concentrations were determined using enzymatic assay kits (DiaSys Diagnostic Systems, Shanghai) on a Roche COBAS c502 analyzer.

### 2.8. Quantitative Real‐Time PCR (qPCR)

Total RNA was extracted using TRIzol reagent (Vazyme, R401) and reverse‐transcribed into cDNA using HiScript II Q RT SuperMix (Vazyme, R222‐01). qPCR was performed on an Applied Biosystems 7500 system with ChamQ SYBR qPCR Master Mix (Vazyme, Q331‐02). Relative expression levels were calculated using the 2^−*ΔΔ*Ct^ method and normalized to *β*‐actin [20]. Primer sequences are listed in Table 1.

**Table 1:** Primers used in this study.

Type	Gene	Forward primer	Reverse primer
Human	*β*‐Actin	5 ^′^CATGTACGTTGCTATCCAGGC3 ^′^	5 ^′^CTCCTTAATGTCACGCACGAT3 ^′^
	HMGCR	5 ^′^TGATTGACCTTTCCAGAGCAAG3 ^′^	5 ^′^CTAAAATTGCCATTCCACGAGC3 ^′^
	HMGCS1	5 ^′^GATGTGGGAATTGTTGCCCTT3 ^′^	5 ^′^ATTGTCTCTGTTCCAACTTCCAG3 ^′^
	DHCR7	5 ^′^GCTGCAAAATCGCAACCCAA3 ^′^	5 ^′^GCTCGCCAGTGAAAACCAGT3 ^′^
	LDLR	5 ^′^TCTGCAACATGGCTAGAGACT3 ^′^	5 ^′^TCCAAGCATTCGTTGGTCCC3 ^′^
	VLDLR	5 ^′^AGAAAAGCCAAATGTGAACCCT3 ^′^	5 ^′^CACTGCCGTCAACACAGTCT3 ^′^
	SCARB1	5 ^′^CCTATCCCCTTCTATCTCTCCG3 ^′^	5 ^′^GGATGTTGGGCATGACGATGT3 ^′^
	ABCA1	5 ^′^ACCCACCCTATGAACAACATGA3 ^′^	5 ^′^GAGTCGGGTAACGGAAACAGG3 ^′^
	ABCG1	5 ^′^ATTCAGGGACCTTTCCTATTCGG3 ^′^	5 ^′^CTCACCACTATTGAACTTCCCG3 ^′^
	APOA1	5 ^′^CCCTGGGATCGAGTGAAGGA3 ^′^	5 ^′^CTGGGACACATAGTCTCTGCC3 ^′^
	CH25H	5 ^′^ATCACCACATACGTGGGCTTT3 ^′^	5 ^′^GTCAGGGTGGATCTTGTAGCG3 ^′^
	CYP7B1	5 ^′^AAAGGTTGGCTTCCTTATCTTGG3 ^′^	5 ^′^GCAACTGACTGATGCTAAATGCT3 ^′^
	CYP7A1	5 ^′^GAGAAGGCAAACGGGTGAAC3 ^′^	5 ^′^GGATTGGCACCAAATTGCAGA3 ^′^
	BCL2	5 ^′^GGTGGGGTCATGTGTGTGG3 ^′^	5 ^′^CGGTTCAGGTACTCAGTCATCC3 ^′^
	PLK4	5 ^′^AAGCTCGACACTTCATGCACC3 ^′^	5 ^′^GCATTTTCAGTTGAGTTGCCAG3 ^′^
	PIK3CA	5 ^′^CCACGACCATCATCAGGTGAA3 ^′^	5 ^′^CCTCACGGAGGCATTCTAAAGT3 ^′^

### 2.9. Immunofluorescence Staining

Cells cultured on coverslips (cell climbing slices) were blocked with 3% BSA and 0.2% Triton X‐100 in PBS for 2 h. The cells were incubated overnight at 4°C with primary antibodies against LDLR (1:100, Abmart, TU378047), ABCA1 (1:100, Abmart, PU115342), CH25H (1:100, Abmart, PS11386), and CYP7B1 (1:100, Abmart, PS10209). Alexa Fluor 594–conjugated secondary antibodies (Thermo Fisher Scientific) were applied, and nuclei were counterstained with DAPI. Images were acquired using an Olympus FSX100 system and analyzed with ImageJ.

### 2.10. Western Blot Analysis

Proteins were extracted from cells cultured in six‐well plates, and concentrations were determined using a BCA assay (Thermo Fisher Scientific). Proteins were separated by SDS‐PAGE and transferred to PVDF membranes (Bio‐Rad). After blocking with 5% BSA, membranes were incubated overnight at 4°C with primary antibodies against CH25H (1:1000, Abmart, PS11386), CYP7B1 (1:1000, Abmart, PS10209), and GAPDH (1:1000, Affinity, T0004). HRP‐conjugated secondary antibodies (Jackson ImmunoResearch) were used, and signals were detected by enhanced chemiluminescence.

### 2.11. Quantitative and Statistical Analysis

Data are presented as mean ± standard deviation (SD). Statistical analyses were performed. Differences among multiple groups were assessed by one‐ or two‐way ANOVA followed by Tukey′s post hoc test. Comparisons between two groups used unpaired two‐tailed Student′s *t*‐tests. Significance levels were set at ∗*p* < 0.05, ∗∗*p* < 0.01, ∗∗∗*p* < 0.001, and ∗∗∗∗*p* < 0.0001. All analyses were conducted using GraphPad Prism 9.0 on Windows 11.

## 3. Results

### 3.1. Metabolic Reprogramming and Cholesterol Pathway Activation in Chemoresistant CRC Cells

To investigate the metabolic basis of chemoresistance, we systematically analyzed transcriptomic profiles of 5‐FU–resistant CRC cell lines using the GSE196900 dataset. Differential expression analysis identified 1175 significantly dysregulated genes (393 upregulated, 782 downregulated; |log2FC| ≥ 1.5, adjusted *p* < 0.05), visualized through a volcano plot (Figure 1a). Functional annotation revealed pronounced enrichment of pathways associated with cytokine/growth factor activity (e.g., TNF signaling) and intercellular communication (e.g., cell adhesion molecules) in resistant cells (Figure 1b,c). Notably, cholesterol metabolism–related processes, including sterol transport regulation and lipid metabolic activation, emerged as key features of the chemoresistant phenotype (Figure 1b,c). Circos plots further mapped critical gene–pathway interactions underlying drug resistance mechanisms (Figure 1d) and cholesterol metabolic rewiring (Figure 1e). PPI network analysis identified functional crosstalk between cholesterol regulators (e.g., ABCA1 and HMGCR) and canonical chemoresistance mediators (e.g., TOP2A and MLH1) (Figure 1f). Complementary GSEA corroborated these findings, showing significant activation of cholesterol‐related biological processes, including cholesterol binding, biosynthesis regulation, and lipid transfer (Figures 1g, 1h, and 1i). Subsequent RNA‐seq analysis revealed decreased cholesterol efflux (ABCA1, ABCG1, and APOE), increased influx (CD36, APOB, and LDLR), enhanced biosynthesis (HMGCR, HMGCS1, and DHCR7), and hydroxylase activity (CH25H, CYP7B1, and Cytochrome P450 Family 7 Subfamily A Member 1 [CYP7A1]) in resistant cell lines, as illustrated in heatmaps depicting differential gene expression (Figure 1j). These multilayered analyses collectively establish hyperactivated cholesterol metabolism as a hallmark of 5‐FU resistance in CRC.

Figure 1RNA‐sequencing analysis unveiled a significant association between cholesterol metabolism and CRC resistance. (a) Volcano plot depicting the variance analysis of differentially expressed genes in GSE196900 (adjusted *p* < 0.05, |Log2FC| ≥ 1.5). (b) Enrichment analysis of GO items in resistant cells. (c) KEGG enrichment of relevant upregulated pathways. Circos plot illustrating underlying genes for significant GO terms containing (d) drug resistance and (e) cholesterol metabolism. (f) PPI network analysis between resistance‐related proteins and cholesterol metabolism–related proteins. GSEA analysis concerning (g) cholesterol binding, (h) regulation of cholesterol biosynthetic process, and (i) cholesterol transfer activity. (j) Heatmaps concerning cholesterol efflux, influx, synthesis and hydroxylase in resistant cells.

**Figure:**
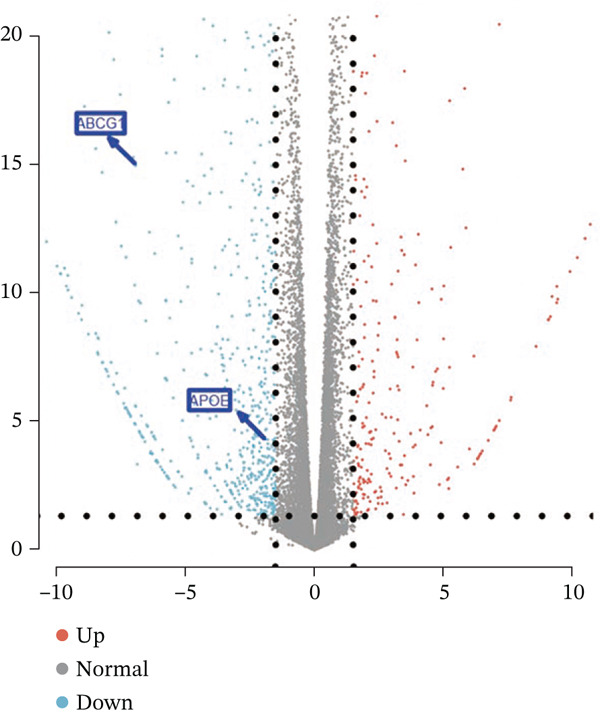
(a)

**Figure:**
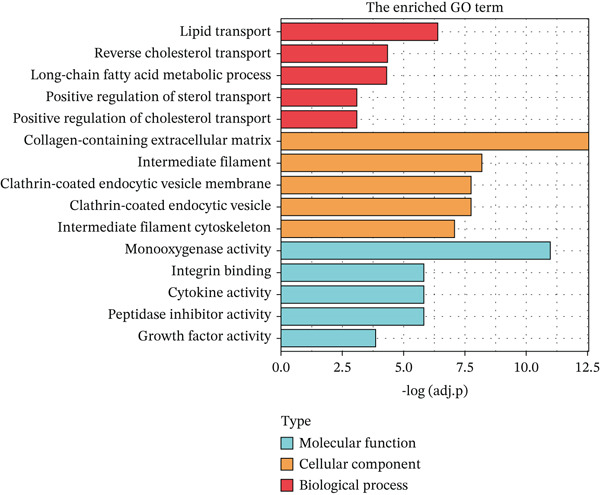
(b)

**Figure:**
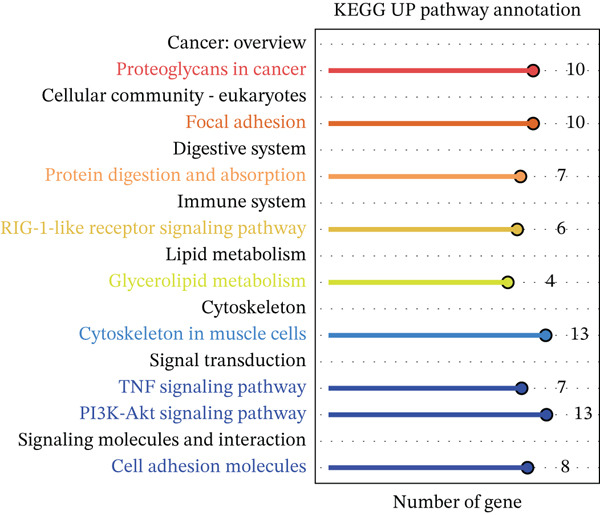
(c)

**Figure:**
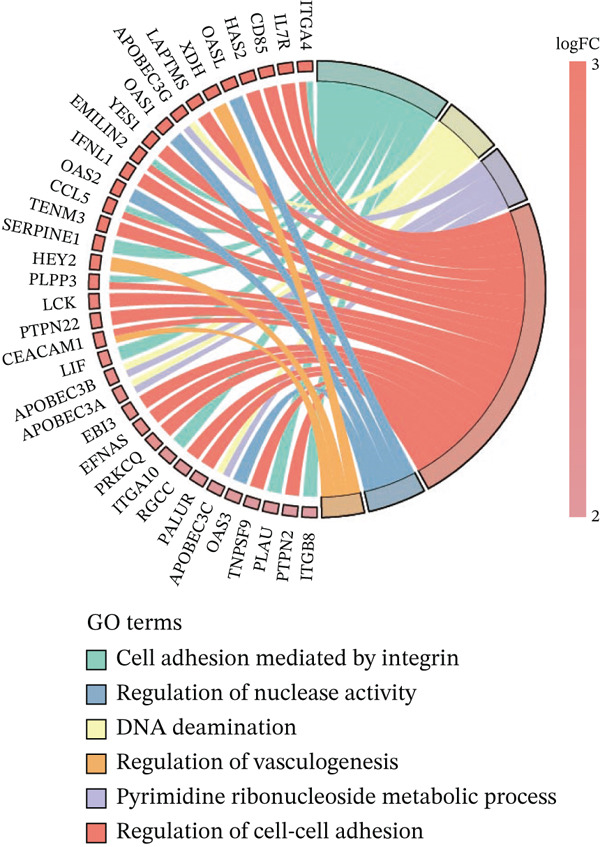
(d)

**Figure:**
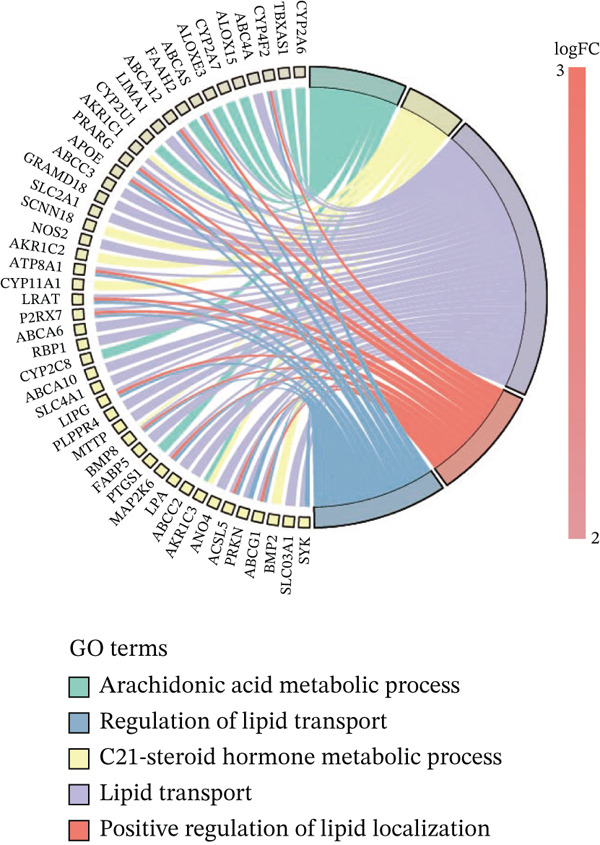
(e)

**Figure:**
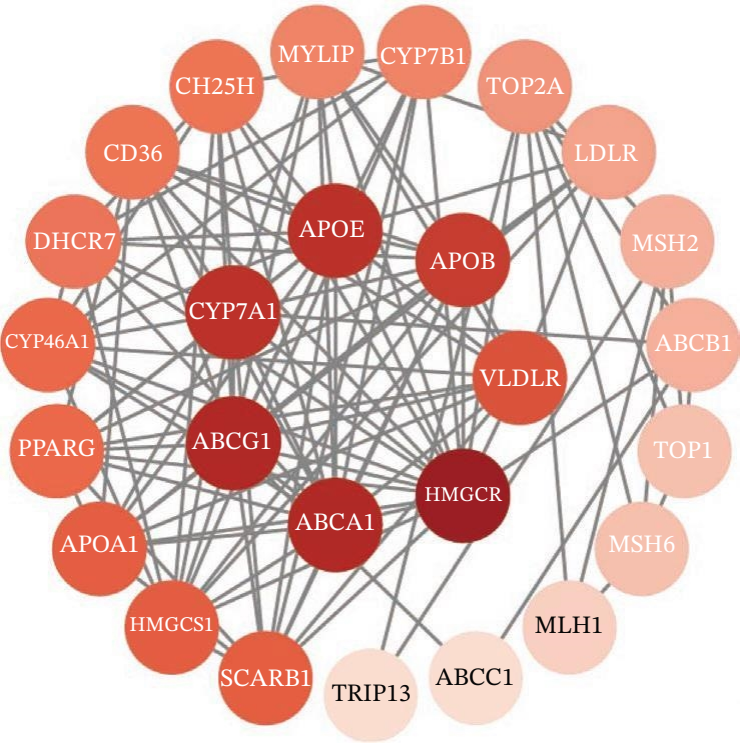
(f)

**Figure:**
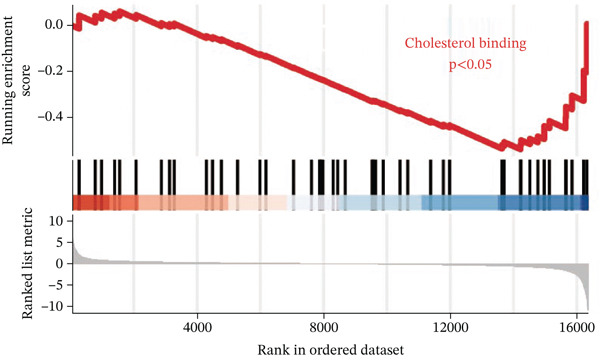
(g)

**Figure:**
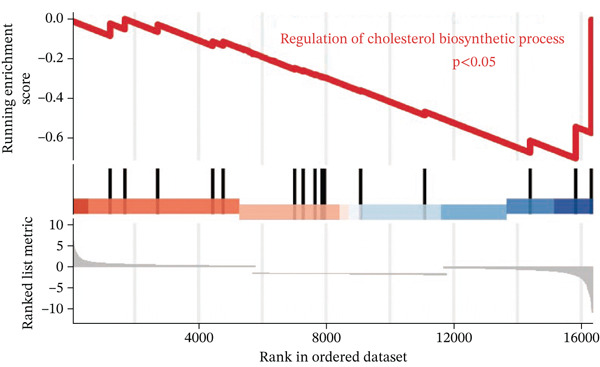
(h)

**Figure:**
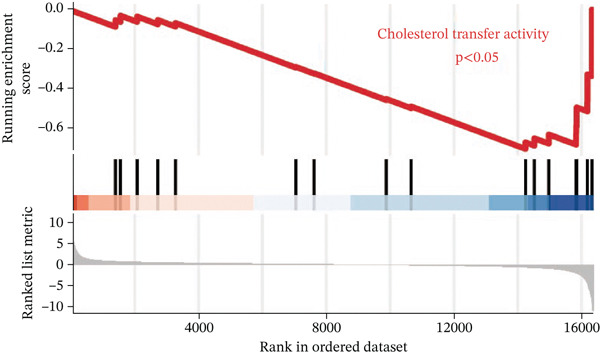
(i)

**Figure:**
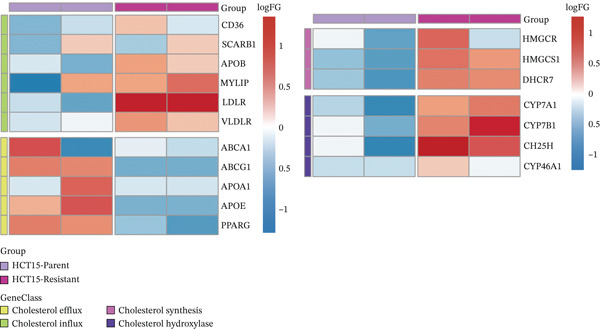
(j)

### 3.2. Metabolic Reprogramming Drives Cholesterol Accumulation in Chemoresistant CRC Cells

In our investigation of cholesterol metabolism in 5‐FU–resistant CRC cell lines, we conducted a comprehensive analysis of gene expression patterns associated with this metabolic pathway (Figure 2a). Consistent with bioinformatics analysis, qPCR revealed significant upregulation of the 3‐hydroxy‐3‐methylglutaryl‐CoA Synthase 1 (HMGCS1) and 7‐dehydrocholesterol reductase (DHCR7) genes in 5‐FU–resistant HCT15 cell lines, indicating an increase in cholesterol biosynthesis. Interestingly, the expression of HMGCR did not show notable changes (Figure 2b). Further qPCR analysis demonstrated upregulation of genes related to cholesterol uptake, including LDLR, very low density lipoprotein receptor (VLDLR), and scavenger receptor class B Member 1 (SCARB1) (Figure 2c). This was corroborated by an increase in LDLR‐positive cells (Figure 2d,e), suggesting an enhanced capacity for cholesterol influx. Conversely, qPCR analysis confirmed downregulation of cholesterol efflux genes such as ABCA1, ABCG1, and apolipoprotein A1 (APOA1) (Figure 2f). Immunostaining assays further validated a reduction in ABCA1 protein levels, a key regulator of cholesterol efflux (Figure 2g,h). This triad of perturbations—amplified synthesis, intensified uptake, and impaired export—culminated in intracellular cholesterol overload, evidenced by 2.1–2.5‐fold increases in total/free/esterified cholesterol and 82% elevated BODIPY fluorescence (Figures 2i, 2j, and 2k). Collectively, these findings mechanistically link cholesterol homeostasis disruption to chemoresistance in CRC.

Figure 2Cholesterol accumulation was revealed in resistant CRC cell lines. (a) Schematic diagram of cholesterol influx and efflux process. (b) qPCR analysis of cholesterol synthesis–related genes HMGCR, HMGCS1, and DHCR7 in 5‐FU–resistant cell lines. (c) qPCR analysis of cholesterol influx–related genes LDLR, VLDLR, and SCARB1. (d) Immunofluorescence assay of LDLR‐positive cells in 5‐FU–resistant cell lines, (e) along with its quantitative analysis (scale bar, 40 *μ*m). (f) qPCR analysis of cholesterol efflux–related genes ABCA1, ABCG1, and APOA1. (g) Immunofluorescence assay of ABCA1‐positive cells in 5‐FU–resistant cell lines, (h) along with its quantitative analysis (scale bar, 40 *μ*m). (i) Measurement of cellular total cholesterol, free cholesterol, and cholesterol ester concentration. (j) BODIPY staining showing cholesterol‐positive cells, (k) along with its quantitative analysis (scale bar, 40 *μ*m). Results are expressed as mean ± SD. The statistical significance of differences ( ^∗^ *p* < 0.05) was assessed by a one‐way or two‐way ANOVA wherever applicable, followed by Tukey′s multiple‐comparisons test.

**Figure:**
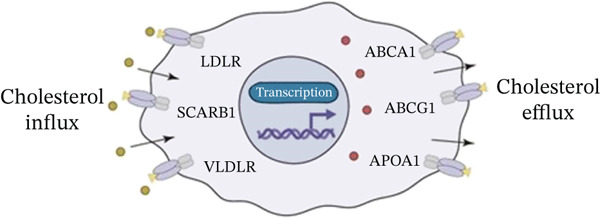
(a)

**Figure:**
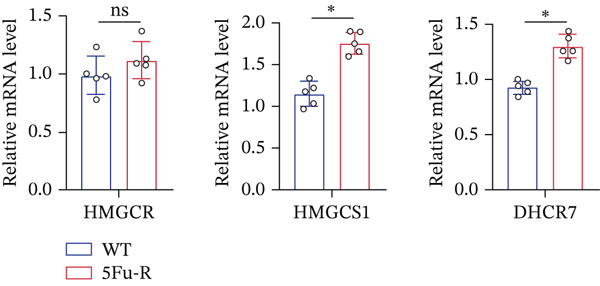
(b)

**Figure:**
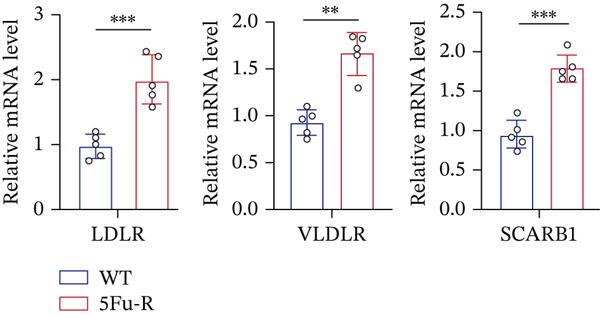
(c)

**Figure:**
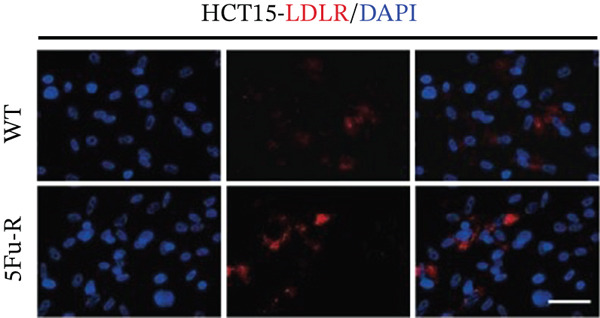
(d)

**Figure:**
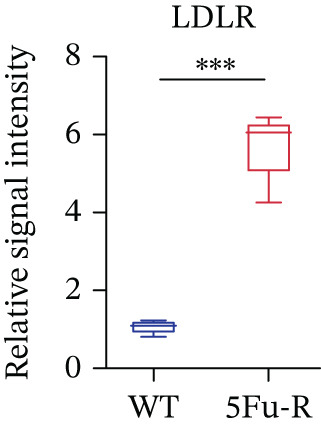
(e)

**Figure:**
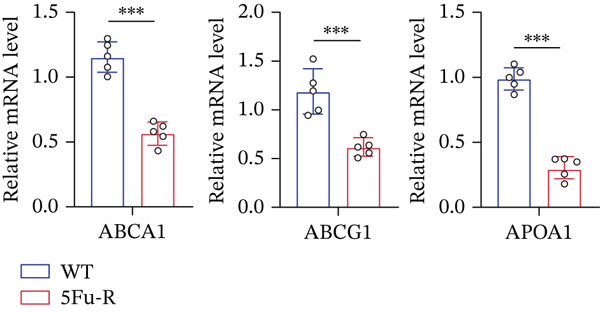
(f)

**Figure:**
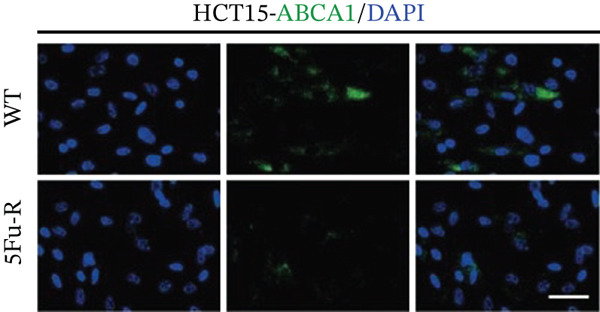
(g)

**Figure:**
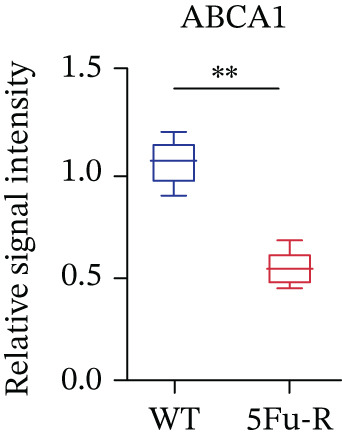
(h)

**Figure:**
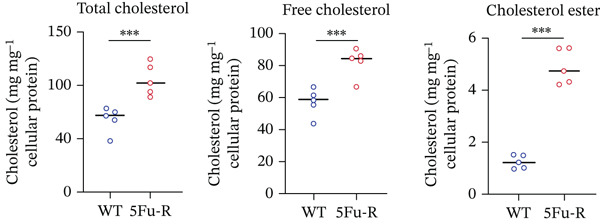
(i)

**Figure:**
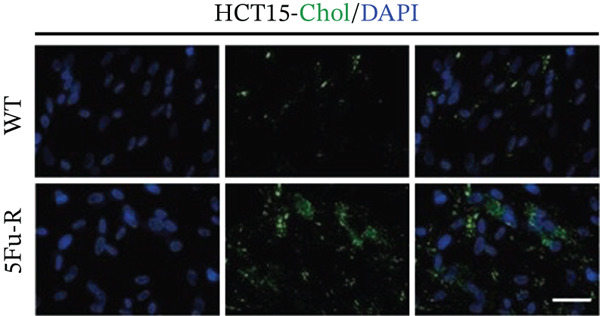
(j)

**Figure:**
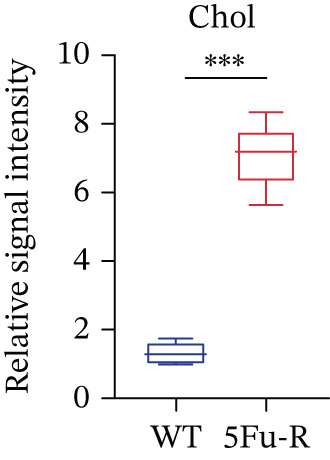
(k)

### 3.3. Cholesterol Drives Chemoresistance Through Proliferative and Migratory Activation in CRC

Systematic interrogation of cholesterol′s functional role revealed its multifaceted protumorigenic effects in CRC models. CCK8 assay demonstrated a significant enhancement of HCT15 cell proliferation in response to cholesterol supplementation, in a time‐ and concentration‐dependent manner (Figure 3a). Notably, a concentration of 100 *μ*M, exerting the most pronounced effect, was selected for subsequent experiments. Further investigation into CRC cell migration was undertaken using the wound‐healing assay. Our findings indicated that cholesterol markedly enhanced the migratory capacity of HCT15 cells (Figure 3b), corroborated by quantitative analysis (Figure 3c). Consistent with these observations, HCT8 cells also demonstrated augmented proliferation and migration in response to cholesterol supplementation (Figures 3d, 3e, and 3f). To determine the effect of cholesterol on chemoresistance, 5‐FU was supplemented at varying concentrations. Cell survival analysis illustrated that cholesterol treatment significantly upregulated the IC_50_ value (2.940 × 10^−6^ M vs. 2.415 × 10^−4^ M; resistance index (RI) = 82.14, 95*%* confidence interval (CI): 78.21–86.33), indicating enhanced drug resistance in HCT15 cells. (Figure 3g). This trend was further confirmed by the observed increase in cell viability under cholesterol supplementation, both with and without 5‐FU administration, after 24 and 48 h (Figure 3h). Consistent mechanophenotypic responses across both cell lines (Figure 3i,j) establish cholesterol as a bona fide mediator of CRC chemoresistance through coordinated activation of proliferation, migration, and drug survival pathways.

Figure 3Cholesterol supplementation augments chemoresistance in CRC cell lines. (a) CCK8 assay of HCT15 treated with cholesterol at different time and concentrations. Data depict one representative experiment of five independent experiments, duplicate conditions for each experiment. (b) Wound‐healing assay of HCT15 treated with cholesterol (100 *μ*M) (scale bar, 125 *μ*m). Data depict one representative experiment of five independent experiments, duplicate conditions for each experiment. (c) Quantitative analysis of aforesaid wound‐healing assay. (d) CCK8 assay of HCT8. (e) Wound‐healing assay of HCT8 (scale bar, 125 *μ*m). (f) Quantitative analysis. (g) Cell counting assay under different doses of 5‐FU for 24 h with or without cholesterol treatment of HCT15 (100 *μ*M). The IC_50_ values in these cells were further calculated with Graphpad Prism 7.0. (h) CCK8 assay of HCT15 treated with or without cholesterol and 5‐FU after 24 h or 48 h. (i) Cell counting assay of HCT8. (j) CCK8 assay of HCT8. Results are expressed as mean ± SD. The statistical significance of differences ( ^∗^ *p* < 0.05) was assessed by a one‐way or two‐way ANOVA wherever applicable, followed by Tukey′s multiple‐comparisons test.

**Figure:**
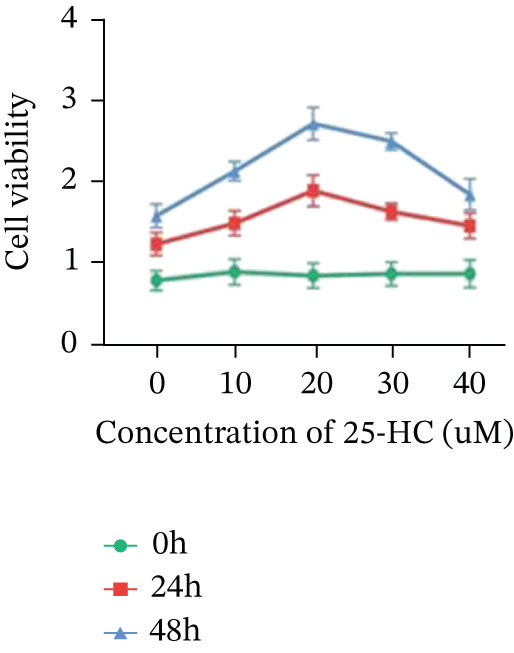
(a)

**Figure:**
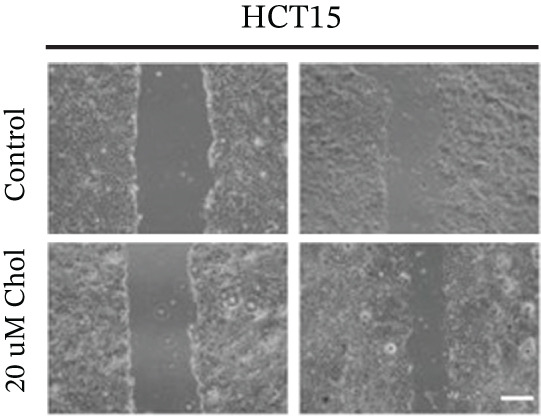
(b)

**Figure:**
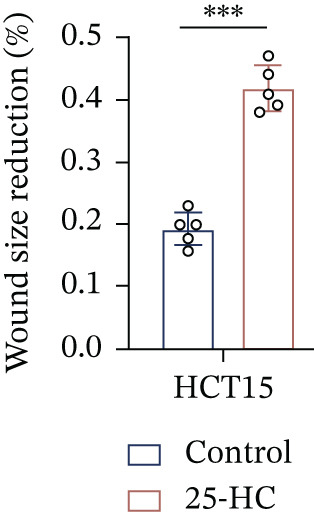
(c)

**Figure:**
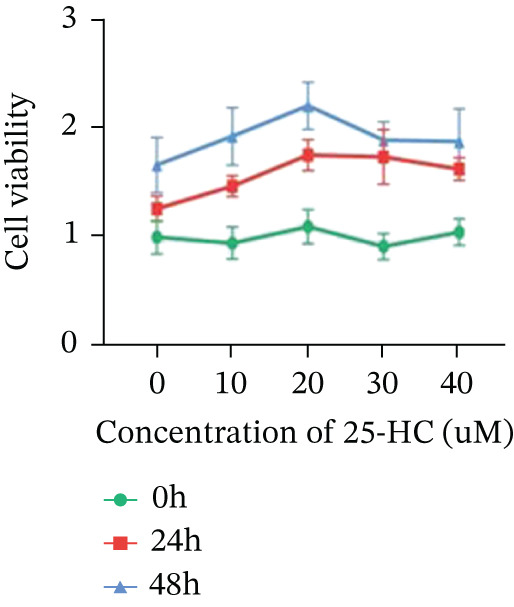
(d)

**Figure:**
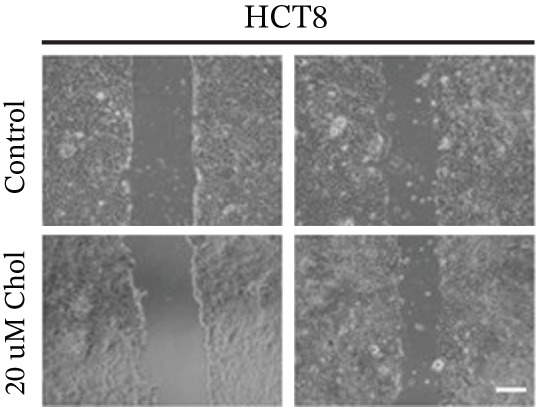
(e)

**Figure:**
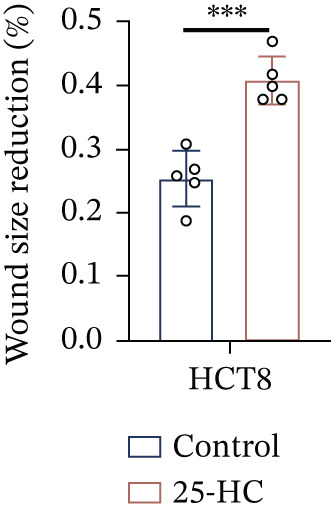
(f)

**Figure:**
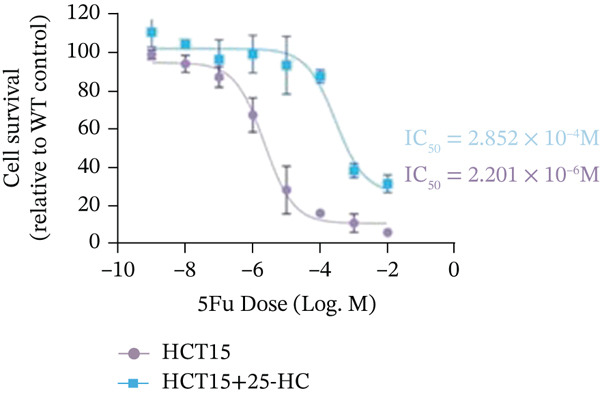
(g)

**Figure:**
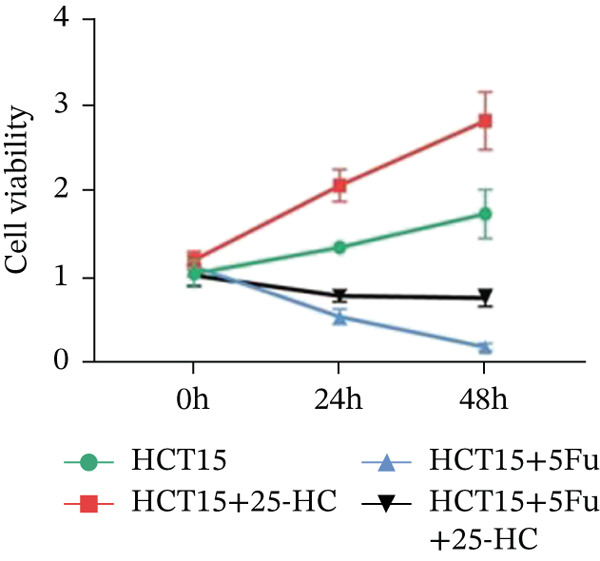
(h)

**Figure:**
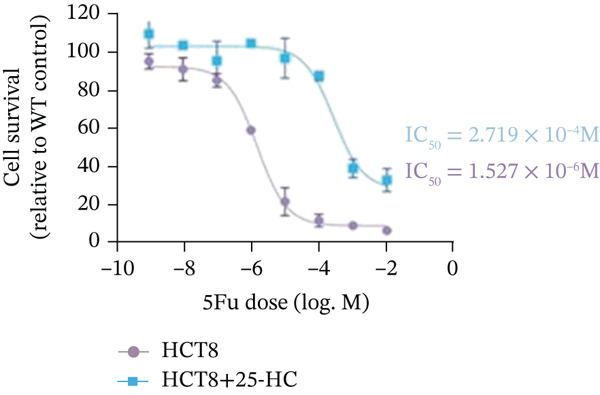
(i)

**Figure:**
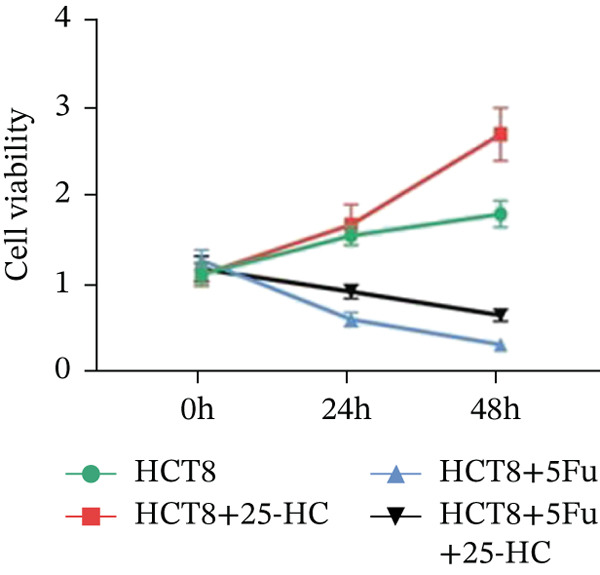
(j)

### 3.4. Cholesterol Potentiates Chemoresistance via Metabolic Rewiring of the CH25H/CYP7B1 Axis

To unravel the molecular mechanisms underlying cholesterol influences on chemoresistance in CRC, we conducted a thorough examination of the underlying downstream metabolic pathways (Figure 4a). Our qPCR analysis in 5‐FU–resistant CRC cells uncovered an upregulation of crucial cholesterol metabolizing enzymes, including CH25H, CYP7A1, and CYP7B1, suggesting an enhanced catabolism of cholesterol contributing to resistance (Figure 4b). Upregulated expression of BCL2 apoptosis regulator, polo like Kinase 4 (PLK4), and phosphatidylinositol‐4,5‐bisphosphate 3‐kinase catalytic subunit alpha (PIK3CA) exerted antiapoptotic, proliferative, and chemoresistance‐promoting effects (Figure 4c). Further immunostaining assay (Figure 4d) and quantitative analysis (Figure 4e) verified an increase in CH25H protein levels. Similar trends were observed for CYP7B1, with a notable rise in the proportion of positive cells (Figure 4f,g). Furthermore, cholesterol supplementation illustrated a concentration‐dependent augmentation in the expression levels of CH25H and CYP7B1. Consistent with previous experiments, a concentration of 100 *μ*M exerts the most significant effect. However, expression of CYP7A1 did not exhibit significant changes (Figure 4h). Additionally, the elevated expression of BCL2, PLK4, and PIK3CA in cholesterol‐supplemented cells further indicated an augmented capacity for antiapoptotic activity, proliferation, and chemoresistance (Figure 4i). Further correlation analysis revealed a positive association between BCL2 expression levels and CH25H (Figure 4j), CYP7B1 (Figure 4k) levels, underscoring the pivotal modulating effect of the CH25H/CYP7B1 axis in chemoresistance. Metabolite 25‐HC catabolized by CH25H from cholesterol also exhibited an increase in 5‐FU–resistant cells (Figure 4l). The downstream product 7*α*,25‐HC metabolized by CYP7B1 also followed a comparable trend (Figure 4m). In cells supplemented with cholesterol, both 25‐HC (Figure 4n) and 7*α*,25‐HC (Figure 4o) exhibited a marked concentration‐dependent increase, with the 100‐*μ*M concentration eliciting the most pronounced effect. Collectively, these results suggest that cholesterol accumulation within CRC cells modulates chemoresistance by activating the CH25H/CYP7B1 axis.

Figure 4Cholesterol accumulation modulated chemoresistance through activating CH25H/CYP7B1 axis. (a) Schematic diagram of cholesterol metabolic pathways. (b) qPCR analysis of cholesterol hydroxylase–related genes CH25H, CYP7A1 and CYP7B1 in 5‐FU–resistant cell lines. (c) qPCR analysis of chemoresistance‐related genes BCL2, PLK4 and PIK3CA. (d) Immunofluorescence assay of CH25H‐positive cells in 5‐FU–resistant cell lines, (e) along with its quantitative analysis (scale bar, 40 *μ*m). (f, g) Immunofluorescence assay of CYP7B1 (scale bar, 40 *μ*m). (h) qPCR analysis of CH25H, CYP7B1, and CYP7A1 genes under different concentrations of cholesterol supplementation. (i) qPCR analysis of BCL2, PLK4, and PIK3CA genes under cholesterol supplementation. Correlation analysis of BCL2 and (j) CH25H, (k) CYP7B1 expression levels. (l) 25‐HC and (m) 7*α*,25‐HC concentration measurement in 5‐FU–resistant cell lines. (n) 25‐HC and (o) 7*α*,25‐HC concentration measurement under different concentrations of cholesterol supplementation. Results are expressed as mean ± SD. The statistical significance of differences ( ^∗^ *p* < 0.05) was assessed by a one‐way or two‐way ANOVA wherever applicable, followed by Tukey′s multiple‐comparisons test.

**Figure:**
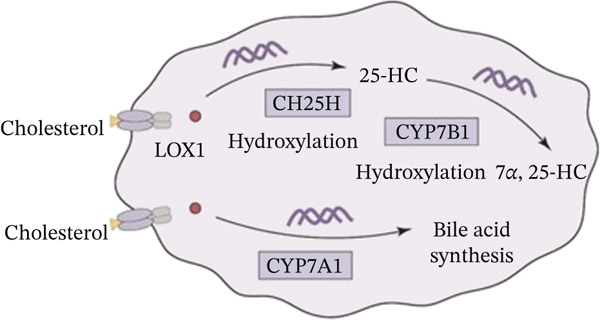
(a)

**Figure:**
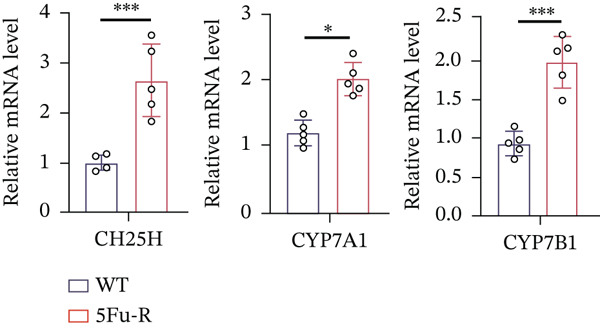
(b)

**Figure:**
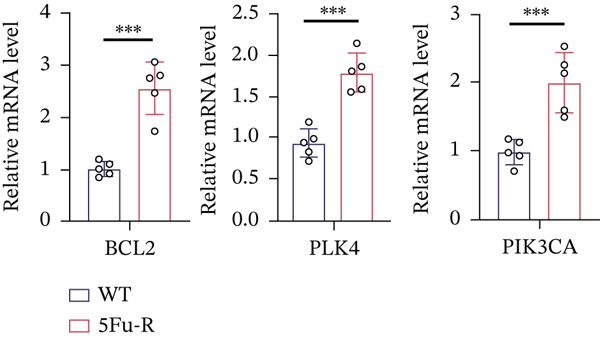
(c)

**Figure:**
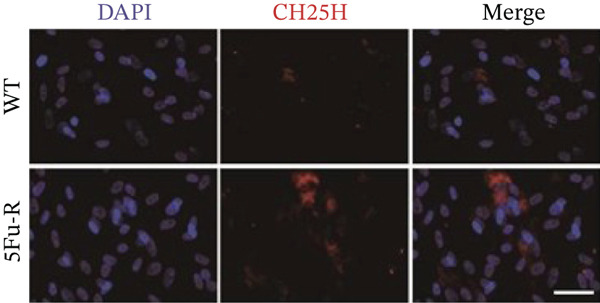
(d)

**Figure:**
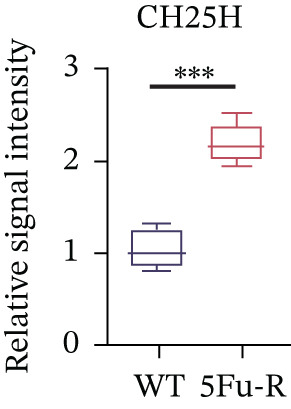
(e)

**Figure:**
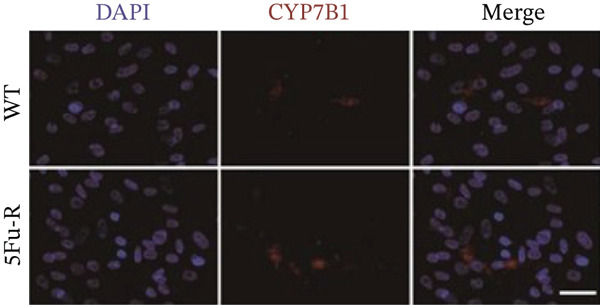
(f)

**Figure:**
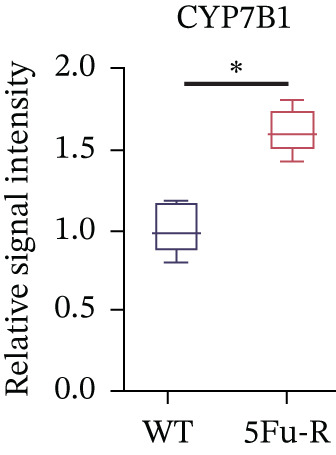
(g)

**Figure:**
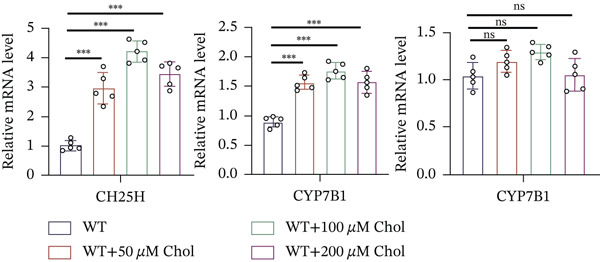
(h)

**Figure:**
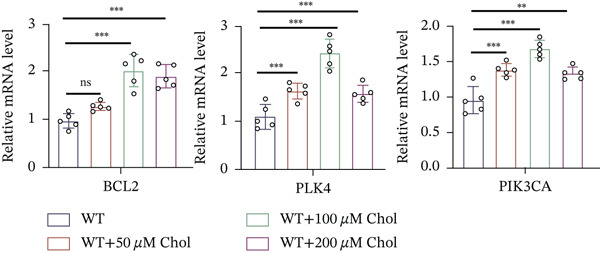
(i)

**Figure:**
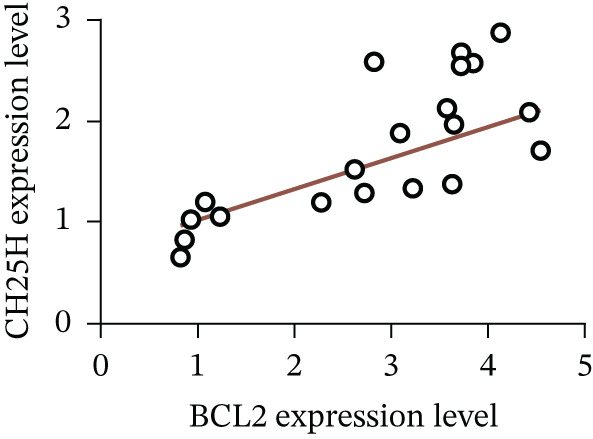
(j)

**Figure:**
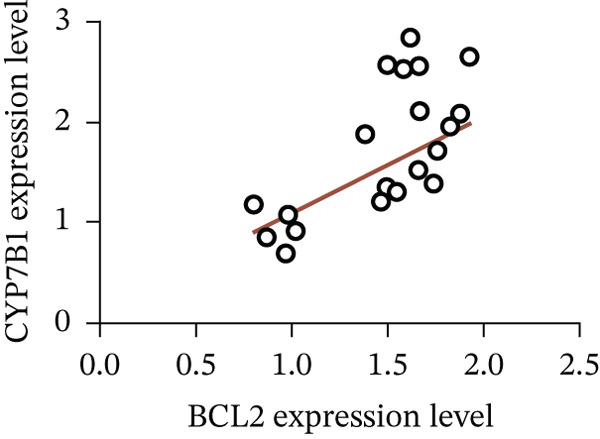
(k)

**Figure:**
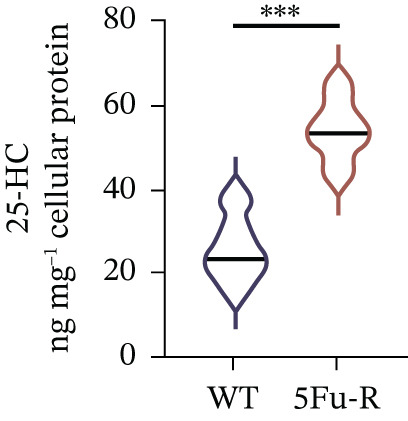
(l)

**Figure:**
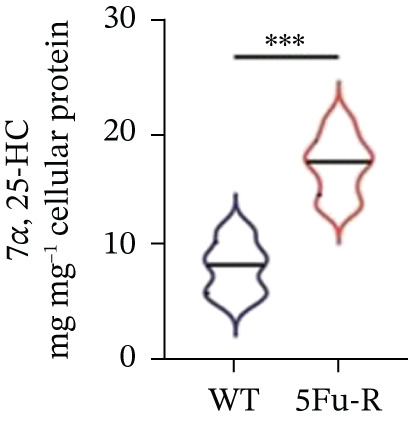
(m)

**Figure:**
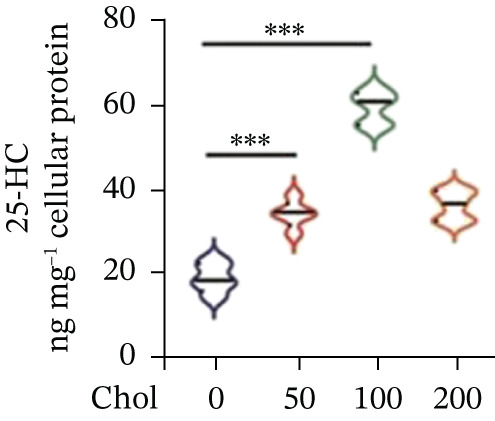
(n)

**Figure:**
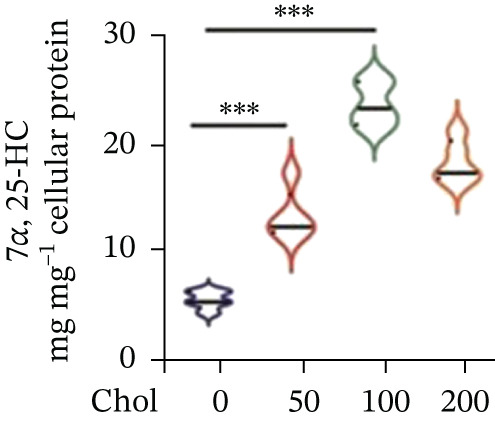
(o)

### 3.5. 25‐HC Mediates Chemoresistance Through Phenotypic Reprogramming in CRC

Building on the established role of cholesterol in activating the downstream CH25H/CYP7B1 axis, we investigated the effects of the cholesterol metabolite 25‐HC, which is catabolized by CH25H. Utilizing the CCK8 assay, we observed a significant enhancement in HCT15 cell proliferation following 25‐HC supplementation, demonstrating a dependence on time and concentration (Figure 5a). Notably, a concentration of 20 *μ*M, yielding the most pronounced effect, was selected for subsequent experiments. Further investigation into CRC cell migration was undertaken using the wound‐healing assay. Our results revealed that 25‐HC substantially enhanced the migratory capacity of HCT15 cells (Figure 5b), corroborated by quantitative analysis (Figure 5c). Consistent with these observations, HCT8 cells also demonstrated enhanced proliferation and migration in response to 25‐HC supplementation (Figures 5d, 5e, and 5f). To ascertain the impact of 25‐HC on chemoresistance, varying concentrations of 5‐FU were subsequently supplemented. Cell survival analysis illustrated that 25‐HC treatment significantly elevated the IC_50_ value (2.201 × 10^−6^ M vs. 2.582 × 10^−4^ M; RI = 82.14, 95% CI: 79.05–85.42), indicating heightened drug resistance in HCT15 cells (Figure 5g). This trend was further validated by the elevated cell viability observed under 25‐HC supplementation, irrespective of 5‐FU treatment after 24 and 48 h (Figure 5h). HCT8 cells responded to 25‐HC in an analogous manner (Figure 5i,j). Collectively, these results mechanistically position 25‐HC as the terminal effector of cholesterol‐mediated chemoresistance, phenocopying parental cholesterol′s effects through enhanced proliferation, metastatic potential, and drug tolerance.

Figure 5Cholesterol downstream 25‐HC supplementation enhanced chemoresistance in CRC. (a) CCK8 assay of HCT15 treated with 25‐HC at different times and concentrations. Data depict one representative experiment of five independent experiments, duplicate conditions for each experiment. (b) Wound‐healing assay of HCT15 treated with 25‐HC (100 *μ*M) (scale bar, 125 *μ*m). Data depict one representative experiment of five independent experiments, duplicate conditions for each experiment. (c) Quantitative analysis of the aforesaid wound‐healing assay. (d) CCK8 assay of HCT8. (e) Wound‐healing assay of HCT8 (scale bar, 125 *μ*m). (f) Quantitative analysis. (g) Cell counting assay under different doses of 5‐FU for 24 h with or without 25‐HC treatment of HCT15 (100 *μ*M). The IC_50_ values in these cells were further calculated with Graphpad Prism 7.0. (h) CCK8 assay of HCT15 treated with or without 25‐HC and 5‐FU after 24 or 48 h. (i) Cell counting assay of HCT8. (j) CCK8 assay of HCT8. Results are expressed as mean ± SD. The statistical significance of differences ( ^∗^ *p* < 0.05) was assessed by a one‐way or two‐way ANOVA wherever applicable, followed by Tukey′s multiple‐comparisons test.

**Figure:**
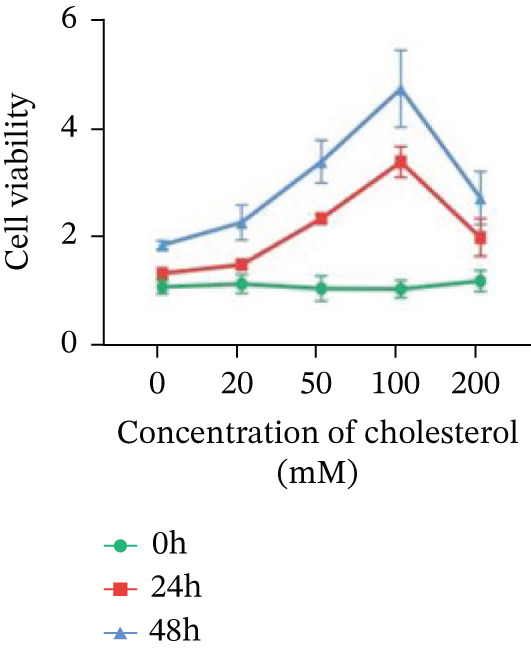
(a)

**Figure:**
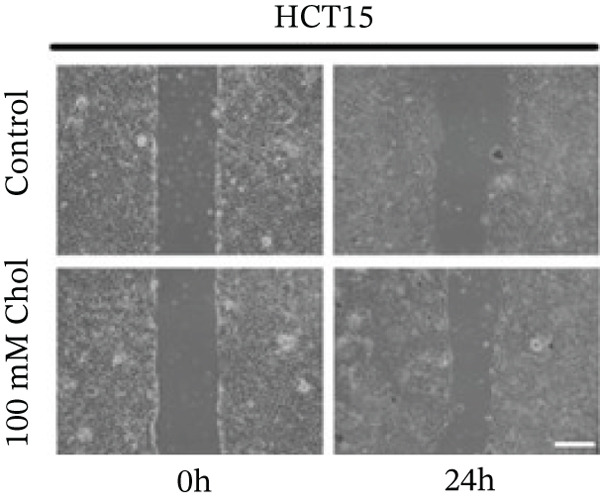
(b)

**Figure:**
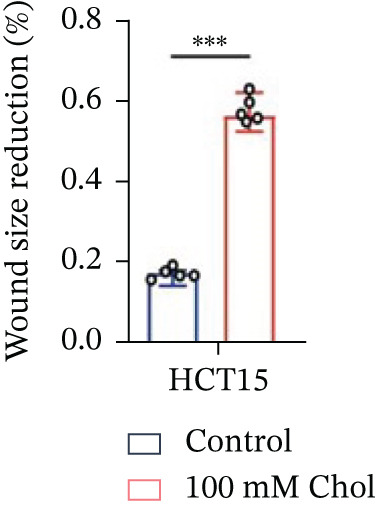
(c)

**Figure:**
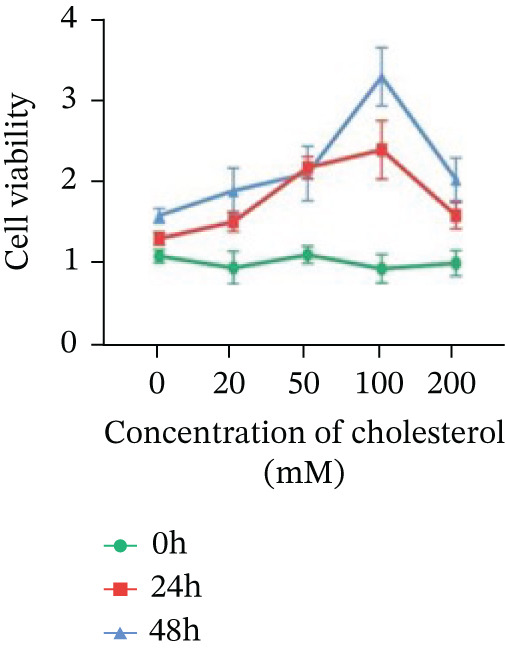
(d)

**Figure:**
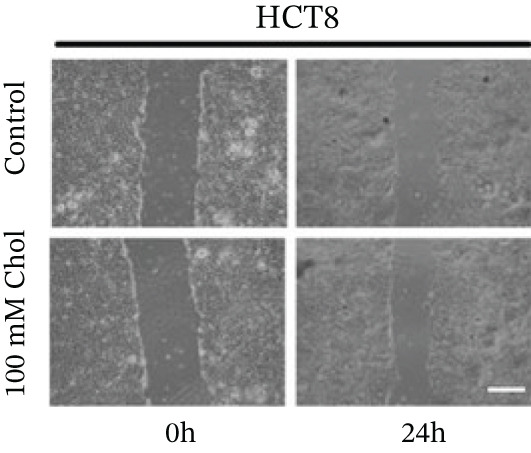
(e)

**Figure:**
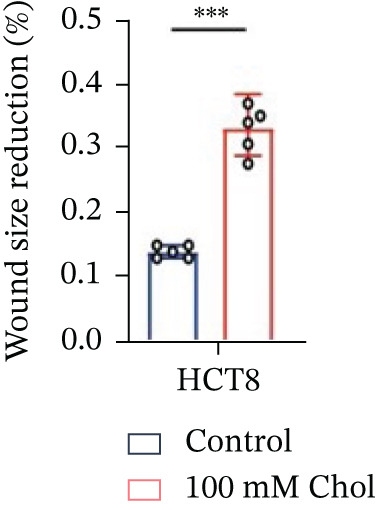
(f)

**Figure:**
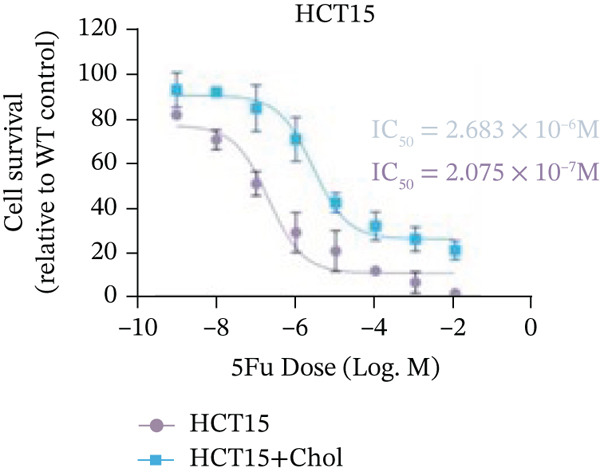
(g)

**Figure:**
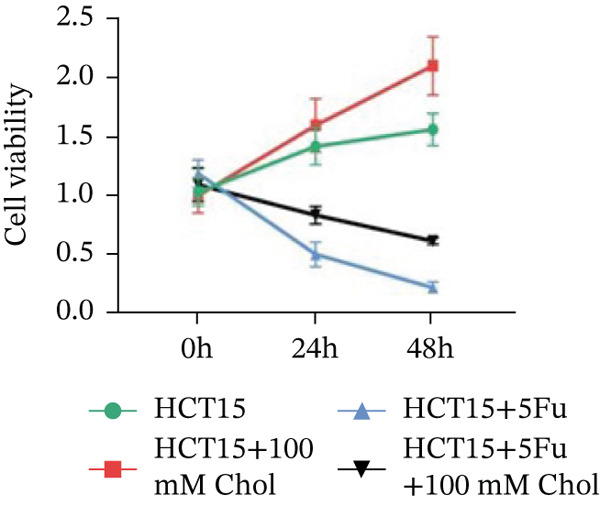
(h)

**Figure:**
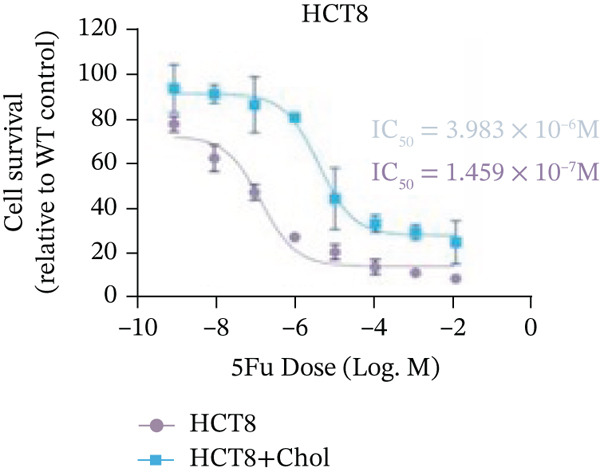
(i)

**Figure:**
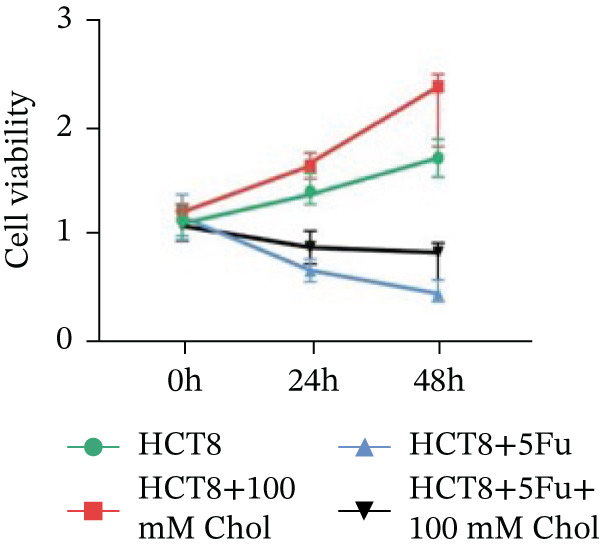
(j)

### 3.6. CH25H Knockdown Abrogates Chemoresistance by Disrupting the CH25H/CYP7B1 Signaling Axis

To explore the specific underlying mechanisms, we used siRNA to target and knock down CH25H. qPCR analysis revealed decreased expression levels of both CH25H and downstream effector CYP7B1 in si‐CH25H treated HCT15 cells, thereby indicating inhibition of the CH25H/CYP7B1 axis (Figure 6a). Western blot further demonstrated a corresponding decrease in protein levels of CH25H and CYP7B1 (Figure 6b), as evidenced by quantitative assessment (Figure 6c). Comparable findings were replicated in HCT8 cells subjected to CH25H knockdown (Figures 6d, 6e, and 6f). CH25H knockdown decreased the IC_50_ value from 2.575 × 10^−6^ M to 1.766 × 10^−7^ M (RI = 14.58, 95% CI: 13.21–16.09), indicating restored 5‐FU sensitivity. This trend was corroborated by a diminished cell viability under CH25H knockdown, irrespective of initial resistance status at both 24 and 48 h post‐treatment (Figures 6g, 6h, and 6i). HCT8 cells mirrored these responses following CH25H knockdown (Figures 6j. 6k, and 6l). Additionally, the diminished expression of BCL2, PLK4, and PIK3CA in CH25H knockdown cells provided additional evidence of a decreased capacity for antiapoptosis, proliferation, and chemoresistance in both HCT15 (Figure 6m) and HCT8 cells (Figure 6n). Although potential off‐target effects of siRNA knockdown could not be completely excluded, the consistent phenotypic rescue observed across multiple assays and independent cell lines (HCT15 and HCT8) upon CH25H silencing supports the specificity of our findings. Together, these findings identify CH25H as a master regulatory node in CRC chemoresistance, whose targeted inhibition resensitizes refractory cells to 5‐FU by dismantling the CH25H/CYP7B1‐BCL2‐PLK4‐PIK3CA survival network.

Figure 6CH25H knockdown blocked CH25H/CYP7B1 axis and inhibited chemoresistance. (a) qPCR analysis of CH25H and CYP7B1 genes under siRNA employment to achieve targeted knockdown of CH25H in HCT15 cells. (b) Western blot analysis of CH25H and CYP7B1 in si‐CH25H treated HCT15 cells, (c) along with quantitative assessment of protein levels. (d–f) qPCR and Western blot analysis in HCT8 cells. (g) Cell counting assay under different doses of 5‐FU for 24 h with or without si‐CH25H treatment of HCT15. The IC_50_ values in these cells were further calculated with Graphpad Prism 7.0. (h) CCK8 assay in si‐CH25H treated resistant HCT15 cells. (i) CCK8 assay in si‐CH25H treated cells under 5‐FU supplementation. (j–l) CCK8 assay of si‐CH25H treated HCT8 cells. (m) qPCR analysis of chemoresistance‐related genes BCL2, PLK4 and PIK3CA in si‐CH25H HCT15 cells. (n) qPCR analysis of BCL2, PLK4 and PIK3CA in si‐CH25H HCT8 cells. Results are expressed as mean ± SD. The statistical significance of differences ( ^∗^ *p* < 0.05) was assessed by a one‐way or two‐way ANOVA wherever applicable, followed by Tukey′s multiple‐comparisons test.

**Figure:**
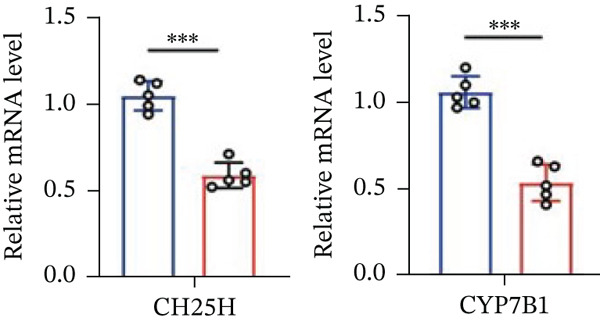
(a)

**Figure:**
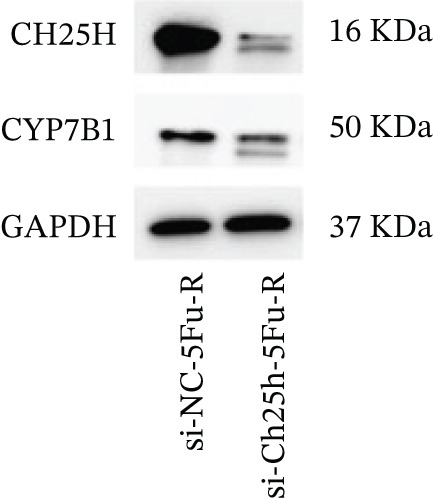
(b)

**Figure:**
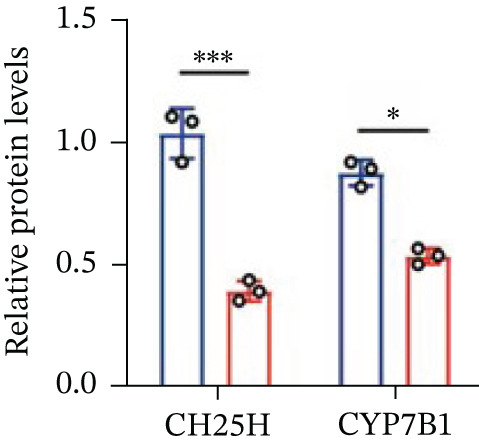
(c)

**Figure:**
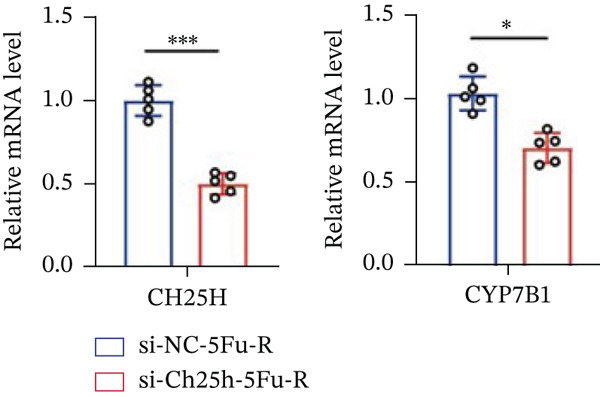
(d)

**Figure:**
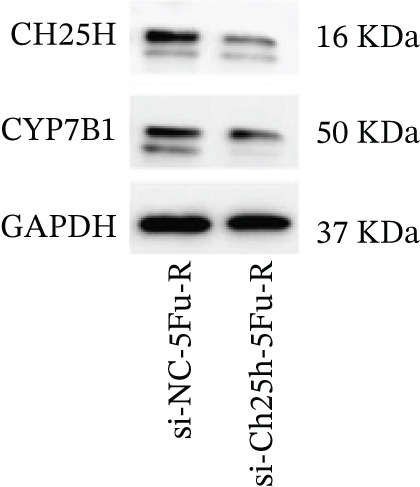
(e)

**Figure:**
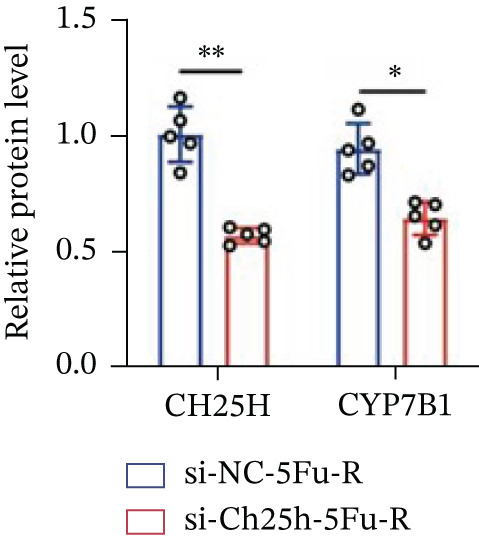
(f)

**Figure:**
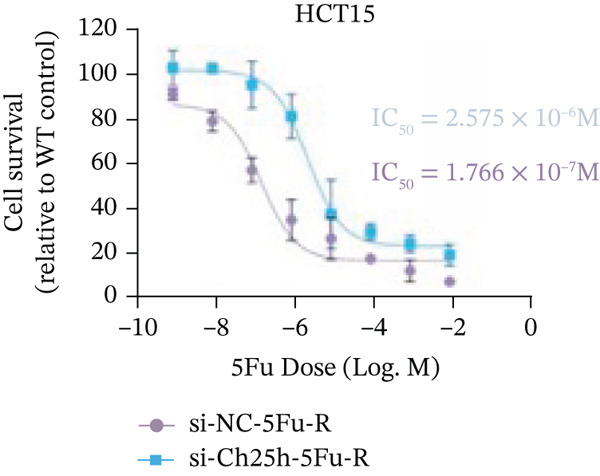
(g)

**Figure:**
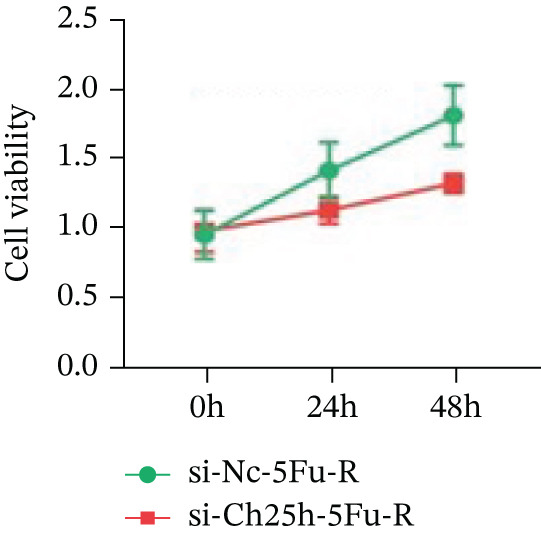
(h)

**Figure:**
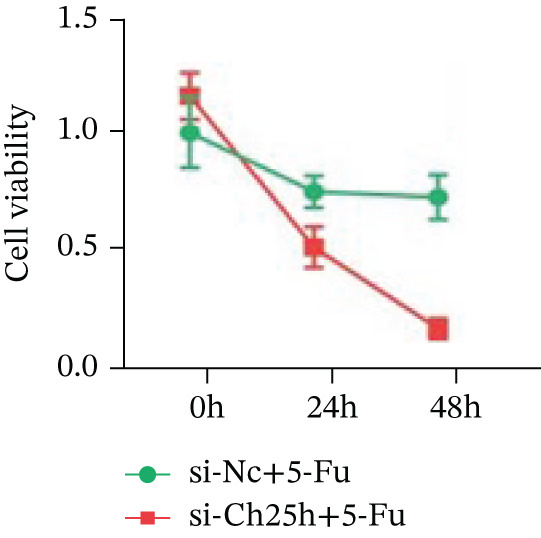
(i)

**Figure:**
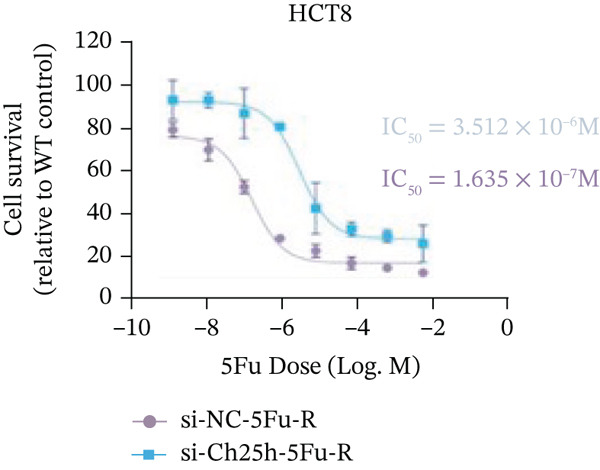
(j)

**Figure:**
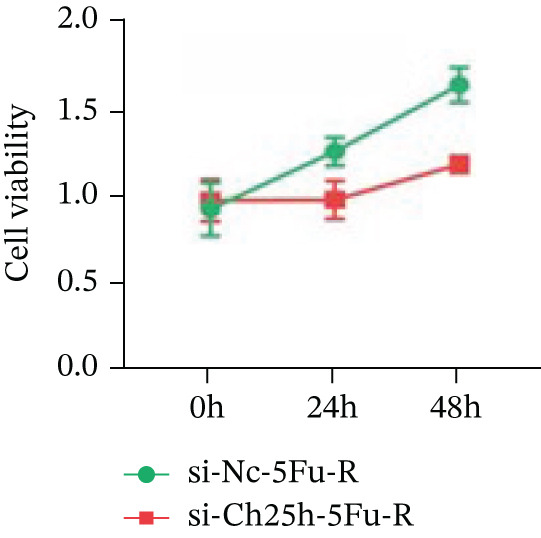
(k)

**Figure:**
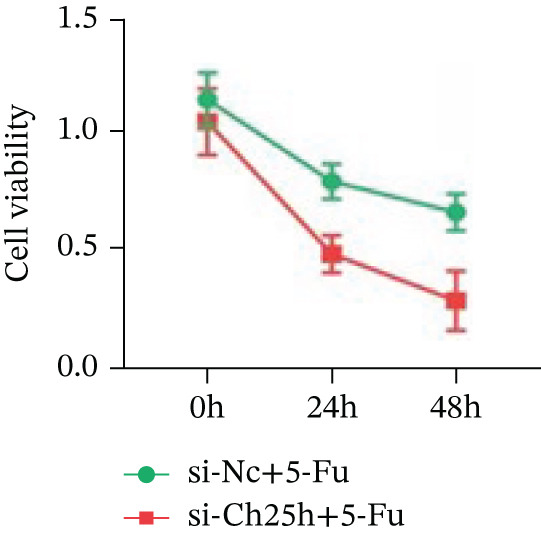
(l)

**Figure:**
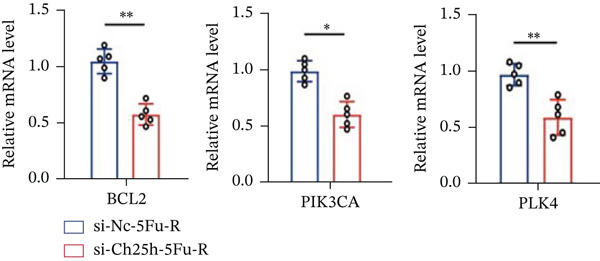
(m)

**Figure:**
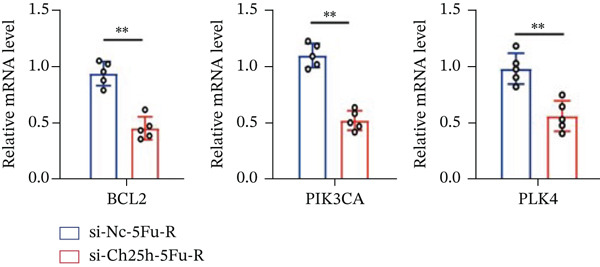
(n)

## 4. Discussion

### 4.1. Metabolic Reprogramming and Cholesterol Dysregulation in CRC

Metabolic reprogramming remains one of the defining cancer hallmarks responsible for supplying essential energy demands for cancer cell proliferation [21]. Although aberrant cholesterol metabolism has been implicated in tumor progression, its mechanistic role in chemoresistance within the CRC microenvironment warrants deeper investigation. In this study, integrative bioinformatics and experimental validation revealed that chemoresistant CRC cells exhibit profound dysregulation of cholesterol homeostasis, characterized by amplified biosynthesis, heightened influx, and compromised efflux pathways. This metabolic rewiring culminates in significant intracellular cholesterol accumulation. Such accumulation further activates the CH25H/CYP7B1 axis and subsequent 25‐HC utilization, contributing to abnormal cellular bioenergetics and chemoresistance development. These findings position cholesterol‐driven pathways as attractive targets for novel cancer therapies. Although our earlier research established that intratumor microbiome‐derived butyrate promotes resistance through activation of the PI3K/AKT signaling pathway [2], the present manuscript uncovers a fundamentally distinct mechanism driven by host cholesterol metabolic reprogramming. Here, we identify the LOX1/CH25H/CYP7B1 signaling axis as a novel cholesterol‐dependent pathway that fuels chemoresistance, highlighting a different layer of metabolic regulation in CRC.

### 4.2. The Multifaceted Role of Cholesterol in Cancer Progression

Cholesterol plays a pivotal role in cell membrane integrity, hormone synthesis, vitamin D generation and bile acid secretion, thereby sustaining essential cellular and systemic functions. Reprogramming of lipid metabolism, particularly upregulated cholesterol uptake and biosynthesis, contributes to uncontrolled cell proliferation [15, 22]. It has been demonstrated that there exists an apparent correlation between cholesterol accumulation and patient outcomes like decreased survival rates and advanced cancer progression [23, 24]. Aberrant cholesterol metabolism has been implicated in the progression of multiple cancers, including hepatocellular carcinoma, glioblastoma, ovarian cancer, and CRC [15, 25]. For instance, cholesterol accumulation promoted NSCLC growth [22], and hepatoma cases exhibited a significant increase in mitochondrial cholesterol content compared with normal tissues [23]. An activated cholesterol biosynthetic program also facilitates the progression of triple‐negative breast cancer [26]. As observed in our investigation, cholesterol undergoes metabolic reprogramming and synthetic upregulation in CRC. These cancer cells actively enhanced the uptake and employment of cholesterol, consequently supporting proliferation and migration. Mechanistically, cholesterol downstream pathways modulate immune responses, ferroptosis, autophagy, tumor cell stemness, and DNA damage responses [27, 28]. Specifically, regulation of immune responses contains inhibiting antigen presentation, modulating immune‐effector cells, and enriching immunosuppressive cells like neutrophils and tumor‐associated macrophages [27]. It has been documented that both genetic and pharmacological interventions targeting cholesterol accumulation decreased the self‐renewal capability and tumorigenic potential in CRC models [29]. Collectively, findings underscore the multifaceted role of cholesterol in the TME.

### 4.3. Cholesterol Metabolism in Drug Resistance Mechanisms

In drug‐resistant cancer cells, investigations have also elucidated aberrant cholesterol pathways [23]. Preclinical studies have highlighted cholesterol reprogramming in conferring drug resistance across a variety of cancer types [30]. For instance, in breast cancer, cells resistant to aromatase inhibitors exhibited activated endogenous cholesterol biosynthesis, resulting in the constitutive activation of estrogen receptor‐*α* [31]. Analogous results have been reported in NSCLC, where gefitinib‐resistant cells displayed elevated cholesterol levels [32]. In enzalutamide‐resistant prostate cancer cell lines, knockdown or inhibition of HMGCR has been demonstrated to resensitize cells to enzalutamide [30]. Consistent with previous studies, our research demonstrated significantly elevated cholesterol levels in drug‐resistant cell lines, as evidenced through bioinformatic and experimental validation. This observed cholesterol accumulation is characterized by enhanced biosynthesis, increased influx, and diminished efflux. Metabolic reprogramming of cholesterol in CRC cells appears to be essential for resistance development, as indicated by augmented proliferation, migration, and chemoresistance under exogenous cholesterol supplementation. Emerging as a promising novel therapeutic strategy for resistance, enhancement of this absorption and biosynthesis is critical for CRC cells utilizing cholesterol to trigger downstream signaling pathways.

### 4.4. The LOX1/CH25H/CYP7B1 Axis as a Novel Therapeutic Target

Previous experiments have demonstrated various mechanisms of 5‐FU resistance, including changes in 5‐FU metabolic enzymes, intratumor heterogeneity, epigenetic modifications, and drug influx and efflux rates [33]. In this study, we unveiled a novel mechanism of 5‐FU resistance in CRC cells, wherein the remodeling of cholesterol metabolism plays a pivotal role in activating the CH25H/CYP7B1 axis and rendering 5‐FU ineffective. Endocytosis of extracellular cholesterol is mediated by lectin‐type oxidized LOX1. Once internalized, intracellular cholesterol is converted to 25‐HC by the CH25H enzyme, which is subsequently metabolized to 7*α*, 25‐HC by CYP7B1 [34]. Prior research has implicated that the CH25H/CYP7B1/ROR*α* axis characterized by cholesterol metabolism is involved in cartilage damage during osteoarthritis progression [35, 36]. CH25H in this metabolic cascade has also been identified as integral in intestinal fibrosis [37]. Extending these findings, our study initially demonstrated a progressive upregulation of CH25H and CYP7B1 following cholesterol exposure, identified as critical regulators underlying cholesterol‐modulated chemoresistance. Subsequent supplementation of the downstream metabolite 25‐HC also exerted facilitated effects on cell proliferation, migration, and viability under 5‐FU exposure. Notably, CH25H knockdown significantly inhibited this facilitating effect, by inhibiting cholesterol downstream signaling pathways. Ensuring a sufficient cholesterol supply employed for the downstream CH25H/CYP7B1 axis may be essential to support CRC progression, thus highlighting a fundamental role of cholesterol in CRC resistance.

### 4.5. Conclusion, Limitations, and Future Perspectives

In conclusion, our study establishes that cholesterol accumulation drives 5‐FU resistance in CRC through the CH25H/CYP7B1 axis. Targeting this pathway presents a promising therapeutic strategy to overcome chemoresistance and resensitize CRC cells to 5‐FU treatment, potentially paving the way for novel personalized therapies based on metabolic profiling.

It is important to note that this study was conducted in monocultured CRC cell lines, which do not fully recapitulate the complexity of the TME. In vivo factors, such as cross talk with CAFs, immune cells, and extracellular matrix components, may significantly modulate the LOX1/CH25H/CYP7B1 axis. For instance, CAFs could secrete lipids or cytokines that augment cholesterol uptake or oxysterol production in cancer cells. Similarly, tumor‐associated macrophages might interact with oxysterol signaling to foster an immunosuppressive niche. Therefore, future investigations employing coculture models, patient‐derived organoids, or in vivo systems will be crucial to validate the pathophysiological role of this axis and explore its broader implications within the TME.

### 4.6. Conclusion

Our research identified accumulated cholesterol in CRC resistant cells resulting from increased biosynthesis and uptake. Exogenous supplementation of cholesterol significantly amplified the proliferation, migration, and 5‐FU resistance of CRC cells by mechanistically modulating CH25H/CYP7B1 axis activation and facilitating metabolite utilization. These results offered a potential therapeutic advantage for targeting cholesterol metabolism to overcome CRC chemoresistance, with further research warranted to translate findings into clinical applications.

**Nomenclature**
25‐HC: 25‐Hydroxycholesterol
5‐FU: 5‐fluorouracil
APOA1: Apolipoprotein A1
BP: biological process
CAFs: cancer‐associated fibroblasts
CC: cellular component
CCK8: Cell Counting Kit‐8
CH25H: Cholesterol 25‐hydroxylase
CI: confidence interval
CRC: colorectal cancer
CYP7A1: Cytochrome P450 Family 7 Subfamily A Member 1
CYP7B1: Cytochrome P450 Family 7 Subfamily B Member 1
DAPI: 4 ^′^,6‐diamidino‐2‐phenylindole
DAVID: Database for Annotation Visualization and Integrated Discovery
DHCR7: 7‐dehydrocholesterol reductase
FBS: fetal bovine serum
GEO: Gene Expression Omnibus
GO: Gene Ontology
GSEA: gene set enrichment analysis
HMGCS1: 3‐hydroxy‐3‐methylglutaryl‐CoA Synthase 1
HRP: horseradish peroxidase
KEGG: Kyoto Encyclopedia of Genes and Genomes
LDLR: low density lipoprotein receptor
LOX1: LDL Receptor 1
MF: molecular function
PLK4: polo like Kinase 4
PPI: protein–protein interaction
PVDF: polyvinylidene fluoride
qRT‐PCR: quantitative real‐time PCR
RI: resistance index
SCARB1: scavenger receptor class B Member 1
SD: standard deviation
siRNA: small interfering RNA
STRING: Search Tool for Retrieval of Interacting Genes
TME: tumor microenvironment
VLDLR: very low density lipoprotein receptor

## Author Contributions

Haixia Cheng: writing—review and editing, conceptualization, methodology, and project administration; Luyao Huang: writing—original draft, project administration, and conceptualization; Jieshen Huang: writing—review and editing, conceptualization, methodology, and project administration; Jiasheng Feng: writing—original draft, conceptualization, and methodology; Kehua Huang: writing—review and editing, conceptualization, methodology, and formal analysis; Mei Liu: writing—review and editing and resources; Ji Li: writing—review and editing and resources. Haixia Cheng, Luyao Huang, and Jieshen Huang contributed equally to this work.

## Funding

No funding was received for this manuscript.

## Disclosure

The results/data/figures in this manuscript have not been published elsewhere, nor are they under consideration by another publisher. All aspects of the work, including the study conception, data collection and analysis, manuscript writing, and editing, were conducted solely by the human authors listed. The content of the manuscript is entirely the original work of the authors, who take full responsibility for its integrity and accuracy. Kehua Huang, Mei Liu, and Ji Li approved the final manuscript.

## Ethics Statement

This study did not involve human participants, human data, or animal experiments. All experiments were conducted using established human colorectal cancer cell lines. Therefore, ethical approval and consent to participate were not required for this research.

## Consent

The authors have nothing to report.

## Conflicts of Interest

The authors declare no conflicts of interests.

## Data Availability

The data that support the findings of this study are available in Gene Expression Omnibus at https://www.ncbi.nlm.nih.gov/geo/, Reference Number GSE196900. All data analyzed during this study are included in this published article.
